# Transcriptomic Analysis of Phenotypic Changes in Birch (*Betula platyphylla*) Autotetraploids

**DOI:** 10.3390/ijms131013012

**Published:** 2012-10-11

**Authors:** Huai-Zhi Mu, Zi-Jia Liu, Lin Lin, Hui-Yu Li, Jing Jiang, Gui-Feng Liu

**Affiliations:** State Key Laboratory of Forest Genetics and Tree Breeding, Northeast Forestry University, 26 Hexing Road, Harbin 150040, China; E-Mails: huaizhimu@gmail.com (H.-Z.M.); zijialiu1987@gmail.com (Z.-J.L.); linlin198212@126.com (L.L.); lihuiyu0519@yahoo.com.cn (H.-Y.L.); jiangjing1960@yahoo.com.cn (J.J.)

**Keywords:** *Betula platyphylla*, autotetraploid, phenotype, transcriptome

## Abstract

Plant breeders have focused much attention on polyploid trees because of their importance to forestry. To evaluate the impact of intraspecies genome duplication on the transcriptome, a series of *Betula platyphylla* autotetraploids and diploids were generated from four full-sib families. The phenotypes and transcriptomes of these autotetraploid individuals were compared with those of diploid trees. Autotetraploids were generally superior in breast-height diameter, volume, leaf, fruit and stoma and were generally inferior in height compared to diploids. Transcriptome data revealed numerous changes in gene expression attributable to autotetraploidization, which resulted in the upregulation of 7052 unigenes and the downregulation of 3658 unigenes. Pathway analysis revealed that the biosynthesis and signal transduction of indoleacetate (IAA) and ethylene were altered after genome duplication, which may have contributed to phenotypic changes. These results shed light on variations in birch autotetraploidization and help identify important genes for the genetic engineering of birch trees.

## 1. Introduction

Polyploidization has played an important role in plant evolution. Many of the phenotypes commonly observed in polyploid derivatives of flowering plants can be grouped together into the “giga” category, which includes increased sizes of floral organs and fruits, larger leaves and often an overall fleshy appearance, making polyploids quite interesting to both agriculture and horticulture [1–3]. In forestry, a greater understanding of the formation and characterization of tree polyploidy has led to well-developed polyploid cultivars. Polyploids in Populus, Robinia, Broussonetia and Edgeworthia have been shown to exhibit such characteristics as greater yield, longer fibers and higher resistance compared to their diploid counterparts [4–10].

The members of the genus Betula form a particularly significant group of broad-leaved trees in Eurasia and North America. Certain birch species, such as *Betula platyphylla*, *Betula pendula*, *Betula pubescens* and *Betula papyrifera*, are valuable sources of wood, and great importance is attached to breeding work aimed at their economic improvement [11]. A natural European birch (*B. verrucosa*) triploid, discovered by Löve, displayed “gigantism” in its morphological organs [12]. Since then, tree-breeding scientists have performed a great deal of work with polyploids in Betula [13–20]. Pieninkeroinen reported that tetraploids in *B. pendula* and *B. pubescens* bear long fibers and vessels [17]. No other studies about artificial polyploid breeding in Betula have been reported, however, and polyploid cultivars in Betula have had not been employed in production. In 2004, 99 *B. platyphylla* autotetraploids were obtained using colchicine treatment. These autotetraploids exhibited phenotypic changes compared to diploids, but information concerning transcriptomic changes after genome duplication in Betula has been scant. In a previous study, researchers often obtained transcriptome profiles of polyploid plants using microarray analysis, which requires whole-genome data [21–23]. Nevertheless, birch is an outbreeding species with a high degree of heterozygosity, the prohibitive costs associated with sequencing and assembling such a complex genome make it unfeasible to consider whole-genome sequencing in the near future. Fortunately, newly developed high-throughput sequencing technology, *i.e.*, next generation sequencing (NGS), including sequencing employing the Roche/454 Genome Sequencer FLX, the ABI SOLiD System and the Illumina Genome Analyzer, is a powerful and cost-efficient tool for advanced research in many areas, especially *de novo* transcriptome sequencing for polyploid plants containing unknown genome information [24,25]. In the present study, we compared differences between transcriptomes of diploid and autotetraploid *B. platyphylla* plants using Illumina paired-end sequencing technology. By investigating changes in the expression of genes attributable to genome duplication, our study deepens our understanding of variations in birch autotetraploidization and identifies important genes for the genetic engineering of birch trees.

## 2. Results

### 2.1. Detection of Birch Autotetraploids

**Figure 1:**
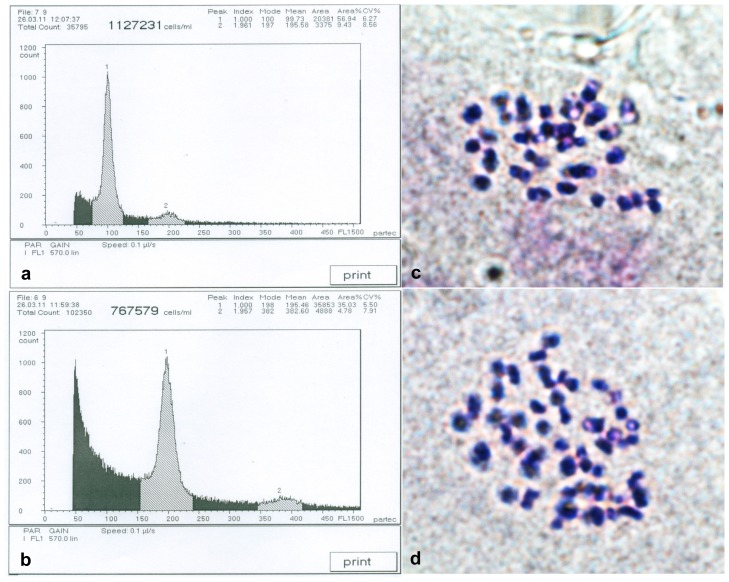
Ploidy analysis of birch saplings. (**a**) DNA content of diploids (the main peak at channel 100); (**b**) DNA content of autotetraploids (the main peak at channel 198). The main peaks represent mature cells and the secondary peaks represent meristematic cells; (**c**) Chromosome number of diploids (2*n* = 2*x* = 28); (**d**) Chromosome number of autotetraploids (2*n* = 4*x* = 56).

Ploidy analysis of novel saplings and control diploid saplings was performed using flow cytometry analysis and chromosome counts. Results from flow cytometry analysis are shown in Figure 1a,b. Figure 1a represents the DNA content of the control diploids, with a main peak at channel 100. Consequently, the saplings with DNA content peaks only at channel 200 were defined as autotetraploids (Figure 1b). The numbers of autotetraploid in the 5 × 3, 5 × 9, 5 × 11 and 6 × 5 families were 31, 22, 23 and 23, respectively, and the total number of autotetraploids was 99.

### 2.2. Phenotypic Changes *versus* Ploidy in Birch

**Figure 2:**
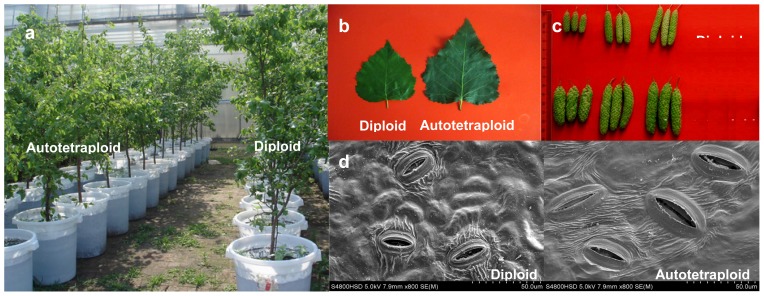
Phenotypic characterization of diploids and autotetraploids grown under the same conditions. (**a**) Diploid and autotetraploid saplings; (**b**) Leaves of diploids and autotetraploids; (**c**) Fruits of diploids and autotetraploids; (**d**) Stomata of diploids and autotetraploids.

The phenotypes of autotetraploid trees differed significantly from diploids in four full-sib families (Figure 2 and Table 1). The breast-height diameter, volume, leaf, fruit and stoma of autotetraploid individuals were significantly larger than those observed in the diploids of each family. The mean breast-height diameter, volume, leaf area, fruit length, fruit diameter and stoma lengths of autotetraploids were 25.09%, 37.31%, 81.43%, 12.37%, 36.97% and 89.72% larger than those of the diploids, respectively. Nevertheless, the heights of the autotetraploid individuals were significantly lower than those observed for the diploids in each family. The mean height of the autotetraploids was only 5.58 m, which was 18.61% lower than that of diploids. The maximal height of the autotetraploid in four families was only 6.51 m, which was 4.96% lower than the mean height observed for the diploids. These results indicate that the vertical growth of the autotetraploids was inferior to that of diploids, but the increase in breast-height diameter and volume and the leaf, fruit and stoma sizes of autotetraploids were superior to those of diploids. Consequently, the autotetraploid trees were stouter, and the diploid trees were more slender.

### 2.3. RNA-Seq, *de Novo* Assembly and Functional Annotation

The apical meristems in plant shoot tips contain a pool of pluripotent stem cells that can self-maintain and produce cells that can differentiate into different cell and tissue types [26], making this tissue a key target for the investigation of plant development and organ formation. Consequently, we constructed a transcriptome from the tender shoot tips of an individual autotetraploid tree and one from its diploid sister from the 6 × 5 family using Illumina paired-end sequencing technology. The transcriptomes of the diploid and autotetraploid produced 4,612,611,600 nt and 4,944,254,580 nt raw data, respectively, and the Q20 percentages were both over 96% (Table 2).

Short reads from two transcriptomic libraries were assembled into unigenes, taking the distance of pair-end reads into account. A library produced from the diploid contained 78,213 unigenes, while a library produced from the autotetraploid contained 88,607 unigenes. The mean lengths of the diploid and autotetraploid unigenes were 625 nt and 622 nt, respectively. All of the sequences were assembled, yielding 84,788 non-redundant unigenes with a mean length of 762 nt, and the N50 was 1294 nt (Table 3).

**Figure 3:**
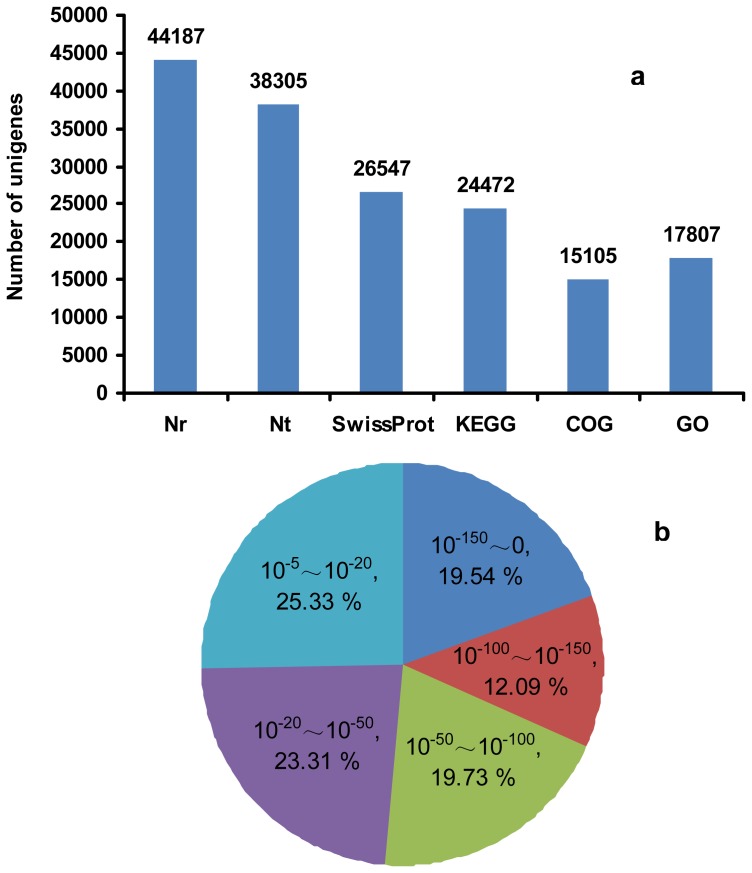
Characteristics of homology-searched birch unigenes. (**a**) Number of unigenes annotated by Blastx and Blastn with an *E*-value threshold of 10^−5^ against the databases. The numbers above the columns indicate the number of unigenes annotated by the databases; (**b**) *E*-value distribution of the top Blastx hits against the Nr database for each unigene.

Approximately 56% of the unigenes (47,324) were annotated by Blastx and Blastn, with a threshold of 10^−5^, to six public databases (Nr, Nt, Swiss-Prot, KEGG, COG and GO). Among them, Unigenes 44,187, 38,305, 26,574, 24,472, 15,105 and 17,807 could be annotated to the Nr, Nt, Swiss-Prot, KEGG, COG and GO database, respectively (Figure 3a). Based on Nr annotation and *E*-value distribution, 74.67% of the mapped sequences showed strong homology (*E*-value ≤ 10^−20^), and 51.36% showed very strong homology (*E*-value ≤ 10^−50^) to available plant sequences (Figure 3b).

### 2.4. Gene Ontology Classfication of DEUs

**Figure 4:**
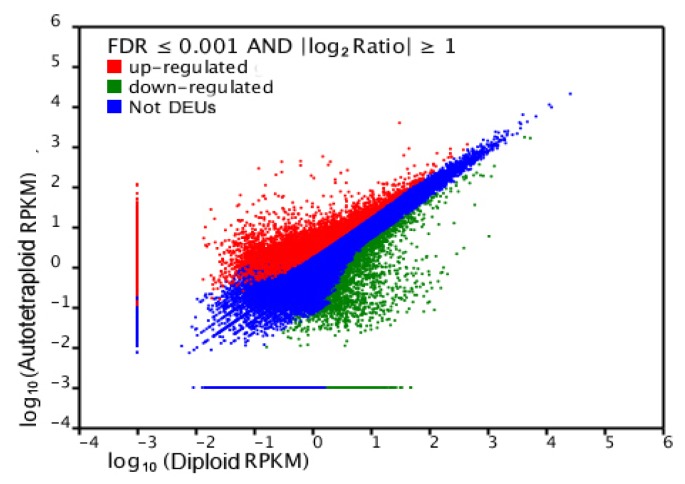
Comparison of unigene expression between autotetraploids and diploids. The differentially expressed unigenes (DEUs) are shown in red and green, while blue indicates unigenes that were not differentially expressed between autotetraploids and diploids.

The differentially expressed unigenes (DEUs) were selected based on expression profiles. A total of 7052 unigenes were found to have been upregulated in the autotetraploids compared to the diploids. Moreover, the expression of 3658 unigenes was downregulated in the autotetraploids (Figure 4).

**Figure 5:**
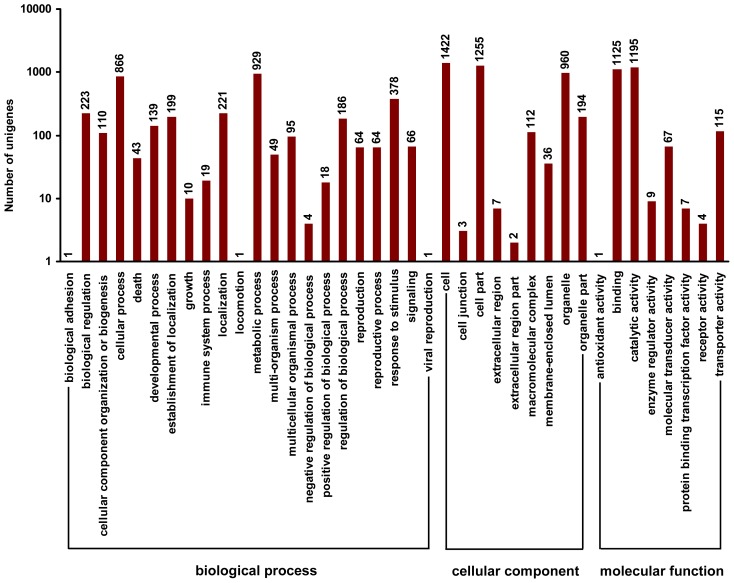
Gene ontology classification of DEUs. The numbers above the columns indicate the numbers of DEUs in each category.

Gene ontology analysis was performed by mapping each DEU to the GO database. In total, 2651 DEUs matched genes in Blast2GO were separated into gene ontology classes according to biological process, cellular component and molecular function (Figure 5). With regard to biological process, metabolic processes (929 unigenes, 35.04%) and cellular processes (866 unigenes, 32.67%) were prominently represented. For cellular component, cell (1422 unigenes, 53.64%), cell part (1255 unigenes, 47.34%) and organelle components (960 unigenes, 36.21%) represented the majorities of this category. Catalytic activity (1195 unigenes, 45.08%) and binding (1125 unigenes, 42.44%) represented a high percentage of the molecular function category. Moreover, cell death, the aminoglycan catabolic process, response to stimulus, the polysaccharide catabolic process and stress differed significantly (corrected *p*-value < 0.05) between autotetraploids and diploids.

### 2.5. Pathway Analysis of DEUs

To further investigate biological behavior, KEGG pathway analysis was used to identify the biological pathways of DEUs. There were 3437 DEUs mapped into 122 KEGG pathways, some of which were consistent with biological processes already revealed by GO analyses. The pathway that differed most significantly between the autotetraploids and diploids was plant-pathogen interactions (Ko04626), followed by stilbenoid, diarylheptanoid and gingerol biosynthesis (Ko00945), limonene and pinene degradation (Ko00903), phenylpropanoid biosynthesis (Ko00940), amino sugar and nucleotide sugar metabolism (Ko00520), flavonoid biosynthesis (Ko00941), biosynthesis of secondary metabolites (Ko01110), plant hormone signal transduction (Ko04075) and phenylalanine metabolism (Ko00360).

**Figure 6:**
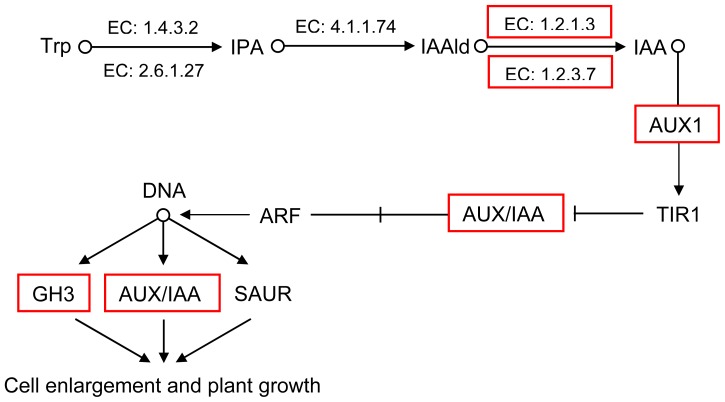
Biosynthesis (IPA pathway) and signal transduction of indoleacetate (IAA) in birch. Up-regulated expressed genes in autotetraploid are in red boxes.

**Figure 7:**
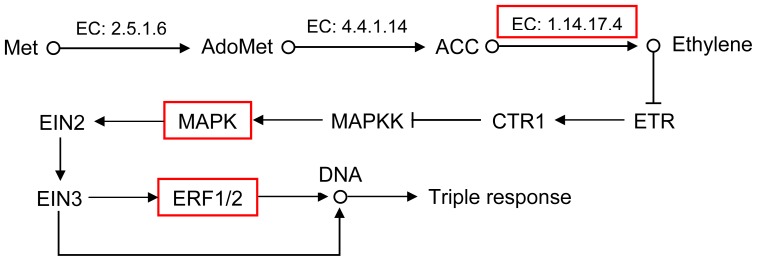
Biosynthesis and signal transduction of ethylene in birch. Up-regulated expressed genes in autotetraploid are in red boxes.

The DEUs could be divided into two groups related to phenotype and transcriptome differences between the autotetraploids and diploids based on previous knowledge about plant growth. The first group of DEUs were involved in the biosynthesis and signal transduction of indoleacetate (IAA), including eleven genes involved in aldehyde dehydrogenase (EC: 1.2.1.3), indole-3-acetaldehyde oxidase (EC: 1.2.3.7), AUX1, AUX/IAA and GH3 (Figure 6 and Table 4). The second group were genes involved in the biosynthesis and signal transduction of ethylene, including six genes involved in aminocyclopropanecarboxylate oxidase (EC: 1.14.17.4), MAPK, ERF1 and ERF2 (Figure 7 and Table 5). All of these genes were upregulated in the autotetraploid.

### 2.6. Verification of RNA-Seq by q-PCR

To test the reliability of RNA-Seq further, q-PCR was performed with specific primers for the 17 differentially expressed genes involved in the biosynthesis and signal transduction of IAA and ethylene.

**Figure 8:**
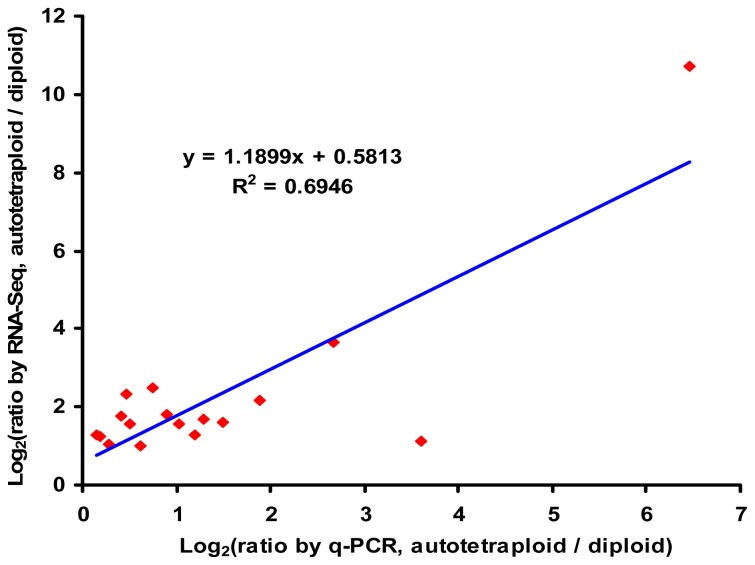
Scatter plot showing similar expression patterns between RNA-Seq and q-PCR of 17 differentially expressed genes involved in the biosynthesis and signal transduction of IAA and ethylene.

Expression patterns revealed by q-PCR analysis were similar to those revealed by RNA-Seq for the same genes (Figure 8). In addition, a significantly positive correlation (*r* = 0.833; *p* < 0.01; *n* = 17) was found between RNA-Seq and q-PCR results from these genes.

## 3. Materials and Methods

### 3.1. Plant Material

The experiment was initiated in April 2004. Seeds from four full-sib families (5 × 3, 5 × 9, 5 × 11 and 6 × 5; all parent plants were located in an intensive seed orchard in Harbin, China) were soaked in 0.1% (*w*/*v*) colchicine for 48 h in the dark. A number of seeds from these four full-sib families were soaked in distilled water for the controls. The seeds were sown in a greenhouse after colchicine treatment, and a total of approximately 7000 saplings were obtained. In 2005, 128 novel saplings and 120 control diploid saplings (30 saplings chosen from each family at random) were transplanted into plastic pots in an intensive seed orchard.

### 3.2. Ploidy Measurement

The DNA contents of leaves of novel saplings were evaluated by flow cytometry (PA, Partec, Germany) using the methods of Zhang *et al*. [27]. Briefly, approximately 2 cm^2^ leaf material was chopped with a razor blade in a plastic petri dish containing 1.5 mL nuclei extraction buffer (10 mmol/L MgSO_4_·7H_2_O, 50 mmol/L KCl, 5 mmol/L HEPES, 1% (*w*/*v*) PVP-40, 0.25% (*v*/*v*) Triton X-100, pH 8.0). The homogenate was filtered through a 30 μm Partec Celltrics™ nylon filter to remove cell debris, and 1 mL staining solution (Partec) was added. The analysis was performed automatically. The total DNA content of each sample was measured, and the percentages of the cells that showed varied DNA content were processed using DPAC software. Meanwhile, the DNA content of diploid leaves was measured in the control group.

Mitotic chromosomes were isolated from young leaf buds using the protoplast dropping method of Anamthawat-Jónsson [28]. Briefly, buds were collected in ice water and treated for 24 h to arrest the cells at metaphase before fixing the buds in a mixture of three parts absolute ethanol to one part glacial acetic acid. The fixed buds were digested in an enzyme mixture of 2.5% (*w*/*v*) cellulase Onozuka R10 (Merck, Darmstadt, Germany) and 2.5% (*v*/*v*) pectinase (Sigma-Aldrich, St. Louis, MO, USA) for 5 h at room temperature. After hypotonic treatment with 75 mmol/L KCl for 5–10 min and repeated fixation to clear the cytoplasm, the protoplasts were placed onto microscope slides using a dropper. The chromosomes were stained with carmine and visualized under a microscope (Axioimanger A1, ZEISS, Germany) using 1000× magnification. Chromosomes were counted from 10 to 20 cells in metaphase from each individual tree.

### 3.3. Phenotype Measurement

Height, breast-height diameter, volume, leaf, fruit and stoma of diploid and autotetraploid trees were measured in 2009. The areas of mature leaves were measured using a portable area meter (LI-3100C, LI-COR, USA). At least ten healthy, undamaged and fully expanded leaves from the top of each individual tree were measured at random.

Stoma lengths were evaluated using a scanning electron microscope (S-4800, Hitachi, Japan). Briefly, approximately 1 cm^2^ from the center of each leaf was fixed in 2.5% (*v*/*v*) glutaral for 24 h and then transferred to 70% (*v*/*v*) ethanol for storage. Prior to evaluation, the material was dehydrated through a graded series of alcohol-isoamyl acetate, critical point dried in carbon dioxide for 15 min with a critical point dryer (HCP-2, Hitachi) and coated with gold palladium at 15 nm with an ion coater (EIKO IB-3, Hitachi). At least 30 stomata from each individual tree were viewed and measured at 15 kV using 800× magnification.

The lengths and diameters of the fruits were measured using a vernier caliper. At least ten healthy, undamaged fruits from each tree that had flowered were selected at random and measured. Height and breast-height diameter measurements were taken after the vegetation period ended. Individual tree volumes were calculated following the methods of Meng [29].

### 3.4. RNA Extraction, Library Construction and RNA-Seq

For transcriptomic analysis, a single autotetraploid tree and its diploid sister from the 6 × 5 family, both of which were healthy, had flowered and possessed erect stems, were chosen. In June 2011, shoot tips from diploid and autotetraploid trees were collected, immediately frozen in liquid nitrogen and stored at −80 °C until use. Total RNA from each sample was isolated using a modified CTAB method and digested with DNaseI (RNase free) to remove contaminating DNA. Enrichment of mRNA, fragment interruption, addition of adapters, size selection and PCR amplification and RNA-Seq were performed by staff at the Beijing Genome Institute (BGI) (Shenzhen, China). Total mRNA was isolated with oligo (dT) cellulose and broken into short fragments. Using these short fragments as templates, the first-strand cDNA and second-strand cDNA were synthesized. Sequencing adapters were ligated to short fragments after purification using a QiaQuick PCR extraction kit, which were used to distinguish different sequencing samples. Fragments that were 200 ± 25 bp in length were then separated by agarose gel electrophoresis and selected as sequencing templates for PCR amplification. Finally, the two transcriptomic libraries were sequenced using Illumina HiSeq™ 2000. The remaining RNA was used for real-time quantitative RT-PCR verification.

### 3.5. *De Novo* Assembly and Functional Annotation

The raw reads were first filtered by removing the adapter sequences and low quality sequences, which included reads with N percentages (*i.e.*, the percentage of nucleotides in a read that could not be sequenced) of over 5% and sequences containing more than 20% nucleotides with a *Q*-value ≤ 10. The *Q*-value represents the sequencing quality of related nucleotides. Clean reads were used in *de novo* assembly and read-mapping to the transcriptome. RNA-Seq data was *de novo* assembled using the Trinity assembling program [30]. For each library, short reads were first assembled into longer but gapless contigs. Then the reads were mapped back to contigs, taking the distance of paired-end reads as frame. The contigs were connected to access the sequence that could not be extended on either end, and the sequence of the unigene was then produced. Next, unigenes from two libraries were further spliced and assembled to obtain maximum length nonredundant unigenes using TGICL clustering software [31] with a minimum overlap length of 100 bp. After clustering, the unigenes were divided to two classes: clusters, with the prefix CL and singletons, with the prefix Unigene. Finally, unigenes were aligned with the NCBI Nr, Swiss-Prot, KEGG and COG protein databases using Blastx with an *E*-value ≤ 10^−5^. The best results were used to further determine the sequence orientations of the unigenes. If results from different databases conflicted with each other, a priority order of Nr, Swiss-Prot, KEGG and COG was followed to determine the sequence orientation. When a unigene did not align with any of the above databases, ESTScan software [32] was used to predict the coding regions and orientation of the sequence. Blast2GO [33] was used to obtain GO annotation of the unigenes based on Blastx hits against the NCBI Nr database. Blastn was used to align the unigenes to the NCBI Nt nucleotide database, retrieving proteins with the highest sequence similarity with the given unigenes along with their protein functional annotations.

### 3.6. Differential Expression of Unigenes

An alignment package, SOAPaligner [34], was used to map reads back to the transcriptome. The number of mapped clean reads for each unigene was counted and normalized into an RPKM value (reads per kb per million reads), which is commonly used to calculate unigene expression [35]. The fold change for each transcript was then calculated using the log_2_ formula of autotetraploid RPKM/diploid RPKM. If the value of either autotetraploid RPKM or diploid RPKM was zero, 0.001, rather than 0, was used to calculate the fold change. The method of Audic *et al*. [36] was used to analyze differential gene expression. The formula used to calculate the probability of a specific gene being expressed equally between the two samples was as follows:

(1) $$P(y|x)={\left(\frac{N_{2}}{N_{1}}\right)}^{y}\frac{(x+y)!}{x!y!(1+\frac{N_{2}}{N_{1}})(x+y+1)}$$

where *N*_1_ and *N*_2_ indicate the total number of clean reads in a diploid and autotetraploid, respectively, and *x* and *y* indicate the mapped clean read counts of the transcript in each sample, respectively. The FDR (false discovery rate) method [37] was used to determine the threshold of *p*-values in multiple tests. In this study, FDR ≤ 0.001 and |log_2_ratio| ≥ 1 were the thresholds employed to judge the significance of differentiated gene expression.

### 3.7. Gene Ontology Functional Enrichment Analysis of Differentially Expressed Unigenes (DEUs)

The analysis mapped all DEUs to GO terms in the database to calculate the numbers of genes for every term. A hypergeometric test was then performed to find significantly enriched GO terms in DEUs compared to the transcriptome background. The formula used for this calculation was as follows:

(2) $$P=1-\sum_{i=0}^{m-1}\frac{\left(\begin{array}{c} M\\ i \end{array}\right)\left(\frac{N-M}{n-i}\right)}{\left(\begin{array}{c} N\\ n \end{array}\right)}$$

where *N* is the number of all unigenes with GO annotation, *n* is the number of DEUs in *N*, *M* is the number of all unigenes annotated to the specific GO terms and *m* is the number of DEUs in *M*. The calculated *p*-value was subjected to Bonferroni Correction using the corrected *p*-value of 0.05 as the threshold. GO terms fulfilling this condition were defined as significantly enriched GO terms in DEUs. This analysis was able to recognize the main biological functions that DEUs exercised.

### 3.8. Pathway Analysis of DEUs

The Blastall program was used to annotate the pathways of DEUs against the KEGG database. The formula used to calculate *p*-value was similar with that used in the GO analysis. In this formula, *N* represents the total number of unigenes with KEGG annotation, *n* is the number of DEUs in *N*, *M* is the total number of unigenes annotated to specific pathways and *m* is the number of DEUs in *M*.

### 3.9. Real-Time Quantitative RT-PCR (q-PCR) Verification

The expression of candidate genes was confirmed by q-PCR. Primers for these genes were designed manually (Table 6). RNA extraction and DNase treatment were performed as described above, and the first-strand cDNA was synthesized using ReverTra qPCR RT Master Mix with gDNA Remover (Toyobo, Osaka, Japan) according to the manufacturer protocol. The cDNA was diluted tenfold and used as the template for q-PCR.

The q-PCR mixture comprised a 3 μL template of the RT reaction mixture, 10 μL of 2× SYBR Green Master Mix (Toyobo, Osaka, Japan) and 0.5 μL of forward and reverse primer (10 μmol/L) brought to a final volume of 20 μL with water. The reactions were performed in an MJ Opticon™^−2^ machine (Bio-Rad, Hercules, CA, USA) using the two-step method that was initiated for 30 s at 94 °C followed by 44 cycles of 94 °C for 12 s, 55 °C for 30 s, 72 °C for 30 s and 78.5 °C for 1 s for plate reading. A melting curve was performed from 55 °C to 95 °C to check the specificity of the amplified product. All experiments were conducted with three biological replicates for each sample. The expression was calculated by 2^−ΔΔCt^ and normalized to values obtained from the *18S* rRNA and *α-tubulin* controls.

## 4. Discussion

Polyploidization events occur frequently during plant evolution and most natural autopolyploids probably did not originate through “simple” genome doubling [38]. For the past few years, analyses of whole-genome sequences, extensive expressed sequence tag (EST) sets and duplicated genomic regions have led to the realization that genome doubling has occurred repeatedly during plant evolution [39]. Variations in plant morphology and physiology resulting from genome duplication have occurred in many plants, which led to the production of fast-growing, high-quality plants due to changes in genes expression that were shown to have occurred [3]. In the present study, we analyzed transcriptomic variation associated with birch (*B. platyphylla*) autotetraploidization that resulted in the differential expression of 10,710 unigenes, some of which are involved in cell death, metabolic processes, plant responses to stimulus and plant hormone signal transduction. These results indicate that phenotypic changes may bear some relation to transcriptomic alteration after genome duplication.

According to transcriptomic analysis, eleven genes were upregulated in the biosynthesis and signal transduction of IAA, including genes involved in aldehyde dehydrogenase, indole-3-acetaldehyde oxidase, AUX1, AUX/IAA and GH3. It has been known for decades that the plant hormone IAA plays a critical role in regulating plant growth and cell enlargement [40]. Although several pathways have been proposed, the indole-3-pyruvic acid (IPA) pathway is the main IAA biosynthetic pathway [41], and IAA is biosynthesized from indole-3-acetaldehyde (IAAld), which is catalyzed by aldehyde dehydrogenase and indole-3-acetaldehyde oxidase [42,43]. During transport, IAA can enter a cell by diffusion or by carrier-mediated uptake and leave a cell through the action of efflux carriers; AUX1 is a major influx carrier [44]. As two related families of proteins, Aux/IAA and ARF are key regulators of IAA-modulated gene expression (AUX/IAA, GH3 and SAUR), and AUX/IAA, GH3 and SAUR play a critical role in regulating plant growth and cell enlargement [40,45]. In the present study, eleven upregulated genes were involved not only in IAA biosynthesis but also in signal transduction. This result provides an explanation for the “gigantism” of breast-height diameter, volume, leaves, fruit and stoma found in autotetraploid trees.

In the present study, it was also shown that the mean breast-height diameter of autotetraploids was 25.09% larger than that of diploids, whereas the mean height of autotetraploids was 18.61% lower than that of diploids. Furthermore, six genes involved in the biosynthesis and signal transduction of ethylene were shown to be upregulated in autotetraploids, including genes involved in the aminocyclopropanecarboxylate oxidase, MAPK, ERF1 and ERF2. Ethylene is a gaseous plant hormone with an effect on seedlings known as the triple response. In the model plant species *Arabidopsis thaliana*, the triple response is characterized by the inhibition of hypocotyl and root elongation, a thickened hypocotyl and an exaggerated apical hook [46]. Ethylene is biosynthesized from 1-aminocyclopropane-1-carboxylate (ACC), which is catalyzed by aminocyclopropanecarboxylate oxidase [47]. MAPK participates in ethylene signal transduction [48]. The transcription factors ERF1 and ERF2 activate the additional downstream ethylene-responsive genes by binding to the GCC-box [49,50]. The six upregulated genes involved in aminocyclopropanecarboxylate oxidase, including MAPK, ERF1 and ERF2, may be related to the stoutness of autotetraploid trees.

Previous research showed that polyploid birch has a higher resistance to biotic and abiotic stress than diploid birch. Triploid birch (*B. verrucosa*× *B. pubescens*) was more resistant to birch rust (*Melampsoridium betulinum*) [14]. Pentaploid and hexaploid birch (*B. papyrifera*) were more tolerant of water deficit than their diploid relatives [20]. This study revealed that responses to stimulus, responses to stress and plant-pathogen interactions differed significantly between autotetraploids and diploids. Therefore, autotetraploid birch (*B. platyphylla*) may have a higher resistance to stress than diploid birch. Consequently, this should be investigated in the future.

Polyploid plants contain new gene regulatory networks after genome duplication, leading to phenotypic and physiological changes [3,39,51]. Complete RNA-Seq data are essential to understanding the new regulatory networks [52]. RNA-Seq analysis of different autotetraploid birch organs and plants in different stages of development should be performed to obtain a deeper understanding of molecular variation after genome duplication.

## Figures and Tables

**Table 1:** Comparison between diploids and autotetraploids in different families with respect to height, breast-height diameter, volume, leaf area, fruit length, fruit diameter and stoma length.

Traits	Full-sib families							

	5 × 3		5 × 9		5 × 11		6 × 5	
			
	Diploid	Autotetraploid	Diploid	Autotetraploid	Diploid	Autotetraploid	Diploid	Autotetraploid
Height (m)	6.80 ± 0.25 A	5.61 ± 0.49 B	6.86 ± 0.17 A	5.70 ± 0.43 B	6.83 ± 0.22 A	5.52 ± 0.48 B	6.92 ± 0.18 A	5.48 ± 0.37 B
Breast-height diameter (mm)	36.44 ± 1.13 B	46.71 ± 3.00 A	36.59 ± 0.91 B	47.30 ± 3.02 A	37.88 ± 1.80 B	46.05 ± 3.35 A	37.59 ± 0.83 B	45.70 ± 3.12 A
Volume (dm^3^)	4.09 ± 0.29 B	5.96 ± 1.07 A	4.15 ± 0.24 B	6.17 ± 1.02 A	4.45 ± 0.52 B	5.74 ± 1.02 A	4.41 ± 0.25 B	5.61 ± 0.91 A
Leaf area (cm^2^)	13.92 ± 5.62 B	28.81 ± 8.63 A	20.77 ± 4.82 B	26.44 ± 5.57 A	13.51 ± 3.87 B	30.64 ± 8.90 A	14.48 ± 3.97 B	27.83 ± 6.40 A
Fruit length (mm)	40.70 ± 2.53 B	48.17 ± 5.25 A	44.51 ± 5.22 B	50.87 ± 6.06 A	45.62 ± 3.59 B	48.53 ± 5.07 A	51.78 ± 6.07 B	57.63 ± 8.39 A
Fruit diameter (mm)	8.55 ± 0.58 B	11.43 ± 2.88 A	8.02 ± 0.53 B	12.76 ± 2.04 A	9.11 ± 0.59 B	12.03 ± 1.19 A	9.02 ± 0.77 B	11.31 ± 1.46 A
Stoma length (μm)	11.09 ± 1.33 B	23.19 ± 5.06 A	16.73 ± 2.04 B	26.31 ± 4.17 A	11.78 ± 1.60 B	27.28 ± 4.64 A	11.84 ± 1.34 B	20.81 ± 3.43 A

Values labeled with different letters differ significantly (*p* < 0.01).

**Table 2:** Throughout and quality of RNA-Seq of diploid and autotetraploid.

Samples	Total reads	Total nucleotides (nt)	Q20 percentage (%)	N percentage (%)	GC percentage (%)
Diploid	51,251,240	4,612,611,600	96.69	0.00	48.53
Autotetraploid	54,936,162	4,944,254,580	96.65	0.00	48.28

Q20 percentage indicates the proportion of nucleotides with quality values higher than 20; N percentage indicates the proportion of unknown nucleotides in clean reads.

**Table 3:** Quality of *de novo* assembly of diploid and autotetraploid.

Samples	Number of unigenes	Total length of unigenes (nt)	Mean length of unigenes (nt)	N50 (nt)
Diploid	78,213	48,907,808	625	1115
Autotetraploid	88,607	55,149,462	622	1117
All	84,788	64,593,413	762	1294

N50 indicates that half of the assembled bases were incorporated into unigenes with a length at least; All indicates nonredundant unigenes assembled by unigenes from diploid and autotetraploid.

**Table 4:** Differentially expressed genes involved in biosynthesis and signal transduction of IAA.

Genes	Log_2_(autotetraploid RPKM/diploid RPKM)	Annotation
GI: 224112551	1.8	auxin influx carrier component
GI: 302398569	1.6	ARF domain class transcription factor
GI: 114733	1.6	RecName: Full = Auxin-induced protein AUX22
GI: 20269055	1.5	AUX/IAA protein
GI: 62125392	1.3	auxin-responsive protein IAA
GI: 302398589	1.3	ARF domain class transcription factor
GI: 42561642	1.2	P-loop containing nucleoside triphosphate hydrolase-like protein
GI: 50404477	1	IAA type protein
GI: 307136360	1.1	auxin-regulated protein
GI: 297802558	1.8	ALDH3I1
GI: 224131694	2.3	aldehyde oxidase 2

**Table 5:** Differentially expressed genes involved in biosynthesis and signal transduction of ethylene.

Genes	Log_2_(autotetraploid RPKM/diploid RPKM)	Annotation
GI: 115345808	2.5	mitogen-activated protein kinase 20
GI: 224123518	10.7	AP2/ERF domain-containing transcription factor
GI: 292668935	3.7	AP2 domain class transcription factor
GI: 289466349	1.7	ERF transcription factor 4
GI: 222427679	2.2	ethylene responsive transcription factor 1a
GI: 62526579	1	ACC oxidase ACCO2

**Table 6:** Primers used for real-time quantitative RT-PCR verification.

Gene-ID	Forward primer (5′-3′)	Reverse primer (5′-3′)	Product size (bp)
*18S rRNA*	ATCTTGGGTTGGGCAGATCG	CATTACTCCGATCCCGAAGG	223
*α-Tubulin*	GCACTGGCCTCCAAGGAT	TGGGTCGCTCAATGTCAAGG	282
Unigene20020_All	AGACTGAGATTTGGGTATCTTCG	CGTTAACAGAACCTGAGCAACC	259
CL26738.Contig1_All	AGACGATGATGATGAATCTTTGC	CCTCAGAAAGATCGACCTAAGG	298
CL37007.Contig1_All	GAACTTGTTCTGAACTGTCTGC	GGAAACTTCAAACGAAGACC	273
Unigene32315_All	GTCTTTCTCCCTCCTTGGTTAGC	TTGTTCTTCCTTAATTTCGTGTCC	273
CL41914.Contig1_All	GCATTGGAAACATTAGAACTGTCC	CCTTCCTTATCGTCAAGCAATCG	264
CL10694.Contig1_All	CCAAGGAACATCTCCTGCAAGC	AGCTCCATCAGCATCAAGGTGG	290
CL1467.Contig1_All	GCTCTGGTTGGTGGAATGG	CCTCAGCTCTGTCTCCTCG	248
CL26579.Contig1_All	CCAGCTGCAAAGACACAAGTGG	TCCAACTAGCATCCAGTCACC	294
CL7645.Contig1_All	GGTTTGAGCAGTTTTGACATAGC	CATCGATGAAGGCAATTCAGTCC	277
CL20989.Contig1_All	GTTCTTGAGAGAGAGAGAGAGG	TACACTGCTTTCGTACGTGTGG	284
Unigene39140_All	TGAGCTCTCCAGTGTCGACC	GGAACCCATCTCTGCTATTTCC	251
Unigene25063_All	CGACCTACTGCAGAAGAGG	TCCTCAAGATATGCAAACTGC	272
Unigene31405_All	CCAACCTTTCTACTATTTTCTGC	GTGGTTTTGGATGAGTTGTTAGC	244
Unigene29640_All	TTGTCGAATCCCTTATCTCAGC	TCACCGGAATCTTTCTCCTGG	265
CL29218.Contig1_All	GGATGAAATCTTTGCAAGCTGG	GCACCAAATCAATGCGTCGAGC	279
CL6280.Contig1_All	TTGGAGCACGAAAGTTTACC	GAGTGGGAAGTTGAGCAAGG	279
CL27664.Contig1_All	CTTCTAGGCAGTGACACTATGG	GTGAACATAGGAGACCAAGTGG	285
